# Atrial Fibrillation in Tribal and Non-tribal Populations: A Comparative Analysis From Eastern India

**DOI:** 10.7759/cureus.117102

**Published:** 2026-09-28

**Authors:** Himanshu Shekhar, Vimukta Pradhan, Jayashree Maity, Pratibha Rai

**Affiliations:** 1 General Medicine, Mahatma Gandhi Memorial Medical College and Hospital, Jamshedpur, IND; 2 Pathology, Mahatma Gandhi Memorial Medical College and Hospital, Jamshedpur, IND; 3 Cardiology, Institute of Medical Sciences, Banaras Hindu University, Varanasi, IND

**Keywords:** atrial fibillation, cha₂ds₂-vasc score, heart failure, left atrial thrombosis, rheumatic heart diseases, tribal population

## Abstract

Background

Atrial fibrillation (AF) is an increasingly prevalent arrhythmia in India, where rheumatic heart disease and cardiometabolic etiologies coexist. This study compared the demographic profile, clinical presentation, associated clinical conditions, echocardiographic characteristics, thromboembolic risk, and anticoagulation practices between tribal patients and non-tribal patients in Eastern India.

Methods

This retrospective hospital-based cross-sectional study included 364 consecutive adult patients with AF (106 tribal and 258 non-tribal patients) admitted between January 2022 and December 2024. Clinical, electrocardiographic, echocardiographic, and treatment data were extracted from hospital records. Continuous variables were compared using Welch's t-test and categorical variables using Pearson's chi-square test.

Results

Tribal patients more frequently presented with first-diagnosed AF (81/106, 76.4% vs. 153/258, 59.3%; p = 0.002), higher ventricular rates (112.6 ± 25.1 vs. 102.3 ± 19.6 beats/min; p < 0.001), lower left ventricular ejection fraction (46.0 ± 11.7% vs. 51.0 ± 10.5%; p < 0.001), and more heart failure symptoms at presentation (27/106, 25.4% vs. 41/258, 15.9%; p = 0.033). Lifestyle-related risk factors were significantly more prevalent among tribal patients (79/106, 74.5% vs. 137/258, 53.1%; p < 0.001). Conversely, non-tribal patients had a higher prevalence of cardiometabolic disorders (168/258, 65.1% vs. 43/106, 40.6%; p < 0.001), ischemic cardiovascular disease (69/258, 26.7% vs. 18/106, 17.0%; p = 0.047), chronic kidney disease (93/258, 36.0% vs. 25/106, 23.6%; p = 0.021), higher CHA₂DS₂-VASc scores (2.38 ± 1.66 vs. 1.86 ± 1.45; p = 0.004), and a greater proportion meeting criteria for long-term oral anticoagulation (192/258, 74.4% vs. 67/106, 63.2%; p = 0.032). Rheumatic heart disease, heart failure, left atrial thrombus, ischemic stroke, and anticoagulant prescribing patterns were comparable between the two groups.

Conclusions

Tribal patients with AF had more first-diagnosed AF, higher ventricular rates, lower left ventricular systolic function, and greater lifestyle-related risk factors, whereas non-tribal patients had greater cardiometabolic and thromboembolic risk. These findings demonstrate differences in the clinical presentation and risk profile of AF within this hospital-based cohort and highlight the need for population-specific strategies to improve AF detection, prevention, and management in Eastern India.

## Introduction

Atrial fibrillation (AF) is a clinically heterogeneous arrhythmia with diverse etiologies, underlying structural cardiac abnormalities, and thromboembolic complications [1]. It is associated with substantial morbidity, mortality, and healthcare utilization and represents a major public health challenge because of its increasing prevalence in recent times [2]. The burden of AF is increasing in India, reflecting the ongoing demographic transition and the rising prevalence of cardiometabolic risk factors [3]. Unlike Western countries, India continues to experience a substantial burden of rheumatic heart disease (RHD) alongside an increasing prevalence of non-rheumatic causes, resulting in a unique clinical and etiological spectrum of AF [4].

Eastern India harbors a large proportion of India’s total tribal population [5]. Scheduled Tribes (STs) predominantly reside in remote regions, where socioeconomic disadvantage and limited access to formal healthcare remain major challenges [5,6]. The tribal population of Eastern India demonstrates distinct health-seeking behaviors, characterized by low health literacy, frequent reliance on traditional remedies, faith healers, and unqualified healthcare providers, often resulting in delayed presentation to healthcare facilities [6]. In addition, sociocultural factors promoting chronic alcohol consumption, the ongoing epidemiological transition toward non-communicable diseases, and the persistent burden of RHD may contribute to distinct clinical characteristics, etiological patterns, and thromboembolic risk profiles of AF among tribal populations compared with the non-tribal population [5,7,8].

Despite several hospital- and community-based studies describing the clinical profile of AF in the general Indian population, comparative data between tribal and non-tribal patients remain scarce [4,9,10]. Whether the distinct demographic, socioeconomic, cultural, and healthcare characteristics of tribal communities translate into differences in their clinical characteristics, underlying etiologies, thromboembolic risks, and anticoagulation practices remains largely unknown. This study primarily aimed to compare the clinical characteristics, etiological spectrum, echocardiographic findings, thromboembolic risk, and anticoagulation practices between tribal and non-tribal patients with AF admitted to a tertiary care hospital in Eastern India.

## Materials and methods

Study design and setting

This retrospective hospital-based cross-sectional comparative observational study was conducted in the Department of General Medicine of a tertiary care teaching hospital in Eastern India. The study aimed to compare the demographic characteristics, clinical presentation, echocardiographic findings, AF-associated clinical conditions, thromboembolic risk profile, and anticoagulation practices between tribal and non-tribal patients with AF.

Study population

Hospital records of all consecutive adult patients (≥18 years) admitted with documented AF on a 12-lead electrocardiogram (ECG) between January 2022 and December 2024 (36 months) were reviewed for eligibility. The AF diagnosis was confirmed by reviewing the medical records together with the corresponding 12-lead ECG available in the case file. Patients with unavailable or insufficient medical records to ascertain the primary study variables, including AF diagnosis, tribal status, clinical characteristics, or echocardiographic findings, were excluded. Patients aged <18 years and those with unconfirmed tribal/non-tribal status were also excluded from the study. To avoid duplicate inclusion, the individual patient was considered the unit of analysis. Duplicate admissions were identified primarily using the unique hospital registration number and further verified by matching the patient's name, date of birth, and telephone number. Only the index hospitalization for each patient was included in the final analysis.

Classification of tribal status

Patients were categorized into tribal and non-tribal groups according to the documented social category recorded in the hospital medical records. Patients recorded as belonging to an ST were classified as tribal, whereas patients with a documented non-ST social category were classified as non-tribal. Patients with missing or unconfirmed social categories were excluded from the study.

Sample size

No formal a priori sample size calculation was performed because this was a retrospective study based on the complete enumeration of all the consecutive eligible patients admitted during a predefined study period. Accordingly, all consecutive eligible patients fulfilling the predefined inclusion and exclusion criteria during the study period were included in the final analysis to minimize selection bias and maximize the available sample size. The final subset comprised 364 patients, which included 106 tribal patients and 258 non-tribal patients.

Data collection

Data were extracted from hospital case records using a pre-designed standardized data extraction proforma specifically developed for the study. Demographic details, clinical presentation, cardiovascular risk factors, comorbidities, laboratory investigations, ECG findings, echocardiographic parameters, associated clinical conditions, thromboembolic risk profile, and anticoagulation therapy were recorded. Data extraction was performed independently by two investigators, and any discrepancies were resolved by consensus after reviewing the original medical records. For variables with missing information, analyses were performed using the available documented data, without imputation of missing values. Variable-specific denominators are therefore reported where applicable. The investigators were not blinded to tribal/ST status during data extraction, as tribal status was obtained from the documented social category in the medical records. As individual patients could present with more than one symptom or clinical manifestation, presenting symptoms were treated as non-mutually exclusive variables and recorded independently.

Definitions

AF was classified as first-diagnosed, paroxysmal, persistent, or permanent according to the 2020 European Society of Cardiology (ESC) Guidelines for the diagnosis and management of AF, based on documented clinical history, previous ECG records, prior hospital documentation, and treating physician documentation available in the medical records [1]. Clinical conditions, cardiovascular risk factors, lifestyle-related risk factors, and comorbidities were identified from documented diagnoses or clinical information available in the hospital medical records, as recorded by the treating physician. Heart failure was defined as a documented diagnosis of heart failure in the hospital medical records, as recorded by the treating physician. Rheumatic heart disease was defined as a documented diagnosis of RHD in the hospital medical records, as recorded by the treating physician and/or supported by echocardiographic findings [1]. When more than one diagnosis or risk factor was documented, each was recorded independently. Cardiometabolic disorders were considered present if hypertension, diabetes mellitus, dyslipidemia, obesity, or metabolic syndrome was documented in the medical records. Ischemic cardiovascular disease included documented coronary artery disease, previous myocardial infarction, previous percutaneous coronary intervention, or previous coronary artery bypass graft surgery. Chronic alcohol consumption was defined as regular or recurrent alcohol use documented in the medical records by the treating physician. Quantitative information regarding the amount and duration of alcohol consumption was not consistently available in this retrospective study.

Echocardiographic assessment

Echocardiographic variables were extracted primarily from transthoracic echocardiography (TTE) reports obtained during hospitalization. TTE was performed in all 364 study subjects. Transesophageal echocardiography (TEE) was performed selectively as an additional investigation when left atrial/left atrial appendage (LA/LAA) thrombus was suspected, TTE visualization was inadequate, or clinical suspicion persisted despite TTE. All documented LA/LAA thrombi were confirmed by TEE. Echocardiographic variables extracted included left atrial diameter, left ventricular ejection fraction (LVEF), left atrial enlargement, RHD, and LA/LAA thrombus.

Thromboembolic risk assessment

Thromboembolic risk was assessed using the CHA₂DS₂-VASc score [11]. Valvular AF was defined according to the 2020 ESC Guidelines as AF in the presence of moderate-to-severe rheumatic mitral stenosis or a mechanical prosthetic heart valve [1]. RHD without moderate-to-severe mitral stenosis was not classified as valvular AF for anticoagulation classification. CHA₂DS₂-VASc scores and eligibility for long-term oral anticoagulation were assessed retrospectively according to the 2020 ESC Guidelines, with oral anticoagulation recommended in men with a CHA₂DS₂-VASc score ≥2 and women with a score ≥3 and considered in men with a score of 1 and women with a score of 2 based on individual assessment by the treating physician. Patients were categorized as meeting guideline-based criteria for anticoagulation [1]. Oral anticoagulant therapy was prescribed at the discretion of the treating physician and was not determined by the study investigators. Patients receiving oral anticoagulation were further categorized according to treatment with vitamin K antagonists (VKAs) or direct oral anticoagulants (DOACs).

Statistical analysis

Data were analyzed using IBM SPSS Statistics for Windows, Version 26.0 (IBM Corp., Armonk, NY, USA). Continuous variables were expressed as mean ± standard deviation (SD), whereas categorical variables were expressed as frequencies and percentages. Comparisons between tribal and non-tribal patients were performed using Welch's independent-samples t-test for continuous variables and the Pearson chi-square test for categorical variables. Welch's test was used for continuous variables because it is robust to unequal variances and unequal sample sizes between groups. Clinical presentation variables, AF-associated clinical conditions, and risk factors were treated as non-mutually exclusive variables because individual patients could present with multiple symptoms, clinical conditions, or risk factors. Each categorical variable was analyzed independently using the Pearson chi-square test. All statistical tests were two-tailed, and a p-value < 0.05 was considered statistically significant.

Ethical considerations

The study protocol was approved by the Institutional Ethics Committee of Rajendra Institute of Medical Sciences (approval 81/IEC, RIMS). Given the retrospective nature of the study and the use of anonymized hospital medical records, the requirement for written informed consent was not applicable. The study was conducted in accordance with the ethical requirements of the Institutional Ethics Committee.

## Results

A total of 364 patients with AF were included in the study, comprising 106 tribal and 258 non-tribal patients. Baseline characteristics were compared using Welch's t-test for continuous variables and the Pearson chi-square test for categorical variables. Demographic characteristics were broadly comparable between tribal and non-tribal patients with AF. The two groups had a similar age and sex distribution, with no significant differences observed in mean age, age categories, or the proportion of male and female patients. Upon presentation, non-tribal patients had significantly higher systolic blood pressure, whereas tribal patients demonstrated a significantly higher mean heart rate (both p < 0.01). A significantly greater proportion of tribal patients presented with first-diagnosed AF compared with non-tribal patients (81/106, 76.4% vs. 153/258, 59.3%; p = 0.002). Persistent AF was the predominant pattern among tribal patients, whereas paroxysmal AF was the most frequent pattern in the non-tribal cohort. Despite these differences in distribution, the overall AF pattern was comparable between the two groups (p = 0.114). The detailed baseline demographic and clinical characteristics of the study population and the comparison between the two study groups are summarized in Table 1.

**Table 1:** Comparison of demographic characteristics and baseline clinical profile between tribal and non-tribal patients with AF Continuous variables are expressed as mean ± standard deviation (SD) and categorical variables as n (%). Group comparisons were performed using Welch's t-test for continuous variables and the Pearson chi-square test for categorical variables. Abbreviations: AF, atrial fibrillation; SD, standard deviation; mmHg, Millimeters of mercury.

Characteristic	Tribal (n=106)	Non-tribal (n=258)	p-value
Age, years (mean ± SD)	56.7 ± 12.8	58.6 ± 16.4	0.27
Age category, n (%)			0.87
≤45 years	24 (22.7)	53 (20.5)	
46-65 years	53 (50.0)	136 (52.7)	
>65 years	29 (27.3)	69 (26.7)	
Systolic blood pressure, mmHg (mean ± SD)	118.5 ± 29.1	128.0 ± 18.2	0.002
Heart rate, beats/min (mean ± SD)	112.6 ± 25.1	102.3 ± 19.6	<0.001
Sex, n (%)			0.75
Male	60 (56.6)	141 (54.7)	
Female	46 (43.4)	117 (45.3)	
AF pattern, n (%)			0.114
Paroxysmal AF	35 (33.1)	115 (44.6)	
Persistent AF	41 (38.7)	87 (33.7)	
Permanent AF	30 (28.3)	56 (21.7)	
First-diagnosed AF, n (%)	81 (76.4)	153 (59.3)	0.002

The spectrum of presenting clinical manifestations was compared between tribal and non-tribal patients with AF. As individual patients could present with more than one symptom or clinical manifestation, each presenting symptom was treated as a non-mutually exclusive binary variable and compared independently between the two groups using Pearson's chi-square test. Palpitations were significantly more common among non-tribal patients (98/258, 38.0%) than tribal patients (27/106, 25.5%; p = 0.022). In contrast, heart failure symptoms, including paroxysmal nocturnal dyspnea, orthopnea, and peripheral edema, were significantly more frequent among tribal patients (27/106, 25.4%) than non-tribal patients (41/258, 15.9%; p = 0.033). Fatigue was the most common presenting symptom in both groups, whereas syncope was the least frequent. No statistically significant differences were observed for dyspnea, chest pain, dizziness/presyncope, syncope, acute ischemic stroke/transient ischemic attack (TIA), or incidentally detected AF. The distribution of presenting clinical manifestations in both study groups is summarized in Table 2.

**Table 2:** Comparison of clinical presentation among tribal and non-tribal patients with AF Values are presented as n (%). Categories are not mutually exclusive because individual patients may have more than one symptom or clinical manifestation at presentation. P-values were calculated using Pearson's chi-square test for each clinical manifestation independently. Abbreviations: AF, atrial fibrillation; TIA, transient ischemic attack. * Heart failure symptoms include paroxysmal nocturnal dyspnea, orthopnea, and/or peripheral edema.

Clinical presentation	Tribal (n=106)	Non-tribal (n=258)	p-value
Fatigue	51 (48.1)	109 (42.2)	0.306
Dyspnea	42 (39.6)	81 (31.4)	0.132
Palpitations	27 (25.5)	98 (38.0)	0.022
Heart failure symptoms*	27 (25.4)	41 (15.9)	0.033
Chest pain	19 (17.9)	33 (12.8)	0.203
Dizziness/presyncope	17 (16.0)	39 (15.1)	0.825
Syncope	4 (3.8)	23 (8.9)	0.089
Acute ischemic stroke/TIA	19 (17.9)	28 (10.9)	0.068
Incidentally detected AF	13 (12.3)	42 (16.3)	0.331

Echocardiographic characteristics were compared using Welch's t-test for continuous variables and the Pearson chi-square test for categorical variables. TTE was performed in all included study subjects. TEE was performed in 27 (25.5%) tribal and 47 (18.2%) non-tribal patients based on indications, including suspected LA/LAA thrombus on TTE, poor visualization of the LA/LAA on TTE, or clinical suspicion of thrombus. All documented LA/LAA thrombi were confirmed by TEE. LA/LAA thrombus was confirmed by TEE in 17 (16.0%) tribal and 26 (10.1%) non-tribal patients. Echocardiographic assessment revealed broadly comparable findings between tribal and non-tribal patients, except for LVEF, which was significantly higher in the non-tribal cohort (51.0 ± 10.5% vs. 46.0 ± 11.7%; p < 0.001). Mean left atrial diameter and the prevalence of left atrial enlargement were similar in both groups. Heart failure and LA/LAA thrombus were observed more frequently among tribal patients, although neither difference reached statistical significance. The prevalence of RHD was also similar in both groups. A detailed comparison of echocardiographic characteristics is shown in Table 3.

**Table 3:** Comparison of echocardiographic characteristics between tribal and non-tribal patients with AF Values are presented as mean ± standard deviation (SD) or n (%). Welch's t-test was used for comparison of continuous variables, whereas categorical variables were compared using the Pearson chi-square test. Abbreviations: AF, atrial fibrillation; SD, standard deviation.

Characteristic	Tribal (n=106)	Non-tribal (n=258)	p-value
Left atrial diameter, mm (mean ± SD)	41.3 ± 5.9	42.4 ± 5.7	0.098
Left atrial enlargement > 40 mm, n (%)	63 (59.4)	137 (53.1)	0.269
Left ventricular ejection fraction, % (mean ± SD)	46.0 ± 11.7	51.0 ± 10.5	<0.001
Heart failure, n (%)	61 (57.5)	123 (47.7)	0.087
Left atrial/left atrial appendage thrombus, n (%)	17 (16.0)	26 (10.1)	0.109
Rheumatic heart disease, n (%)	36 (34.0)	79 (30.6)	0.533

Comparison of AF-associated clinical conditions and risk factors using Pearson's chi-square test demonstrated distinct differences between the two study populations. Tribal patients had a significantly higher prevalence of lifestyle-related risk factors, including chronic alcohol consumption, smoking, smokeless tobacco use, and intravenous drug abuse (79/106, 74.5% vs. 137/258, 53.1%; p < 0.001). In contrast, non-tribal patients had a significantly higher burden of cardiometabolic disorders, ischemic cardiovascular disease, and chronic kidney disease (CKD) (all p < 0.05). Although documented heart failure was numerically more frequent among tribal patients (61/106, 57.5% vs. 123/258, 47.7%), the difference did not reach statistical significance (p = 0.087). The prevalence of RHD and hyperthyroidism did not differ significantly between the two groups. The distribution of AF-associated clinical conditions and risk factors in the two study groups is presented in Table 4.

**Table 4:** Comparison of AF-associated clinical conditions and risk factors between tribal and non-tribal patients Values are presented as n (%). Data are not mutually exclusive because of the overlapping risk factors in the same patient. P-values were calculated using the Pearson chi-square test. *Cardiometabolic disorders were defined as the presence of one or more of the following: hypertension, diabetes mellitus, dyslipidemia, obesity, or metabolic syndrome. † Lifestyle-related risk factors comprised chronic alcohol consumption, smoking, smokeless tobacco use, and substance abuse. Abbreviation: AF, atrial fibrillation

Associated clinical conditions	Tribal (n=106)	Non-tribal (n=258)	p-value
Rheumatic heart disease	36 (34.0)	79 (30.6)	0.533
Heart failure	61 (57.5)	123 (47.7)	0.087
Cardiometabolic disorders*	43 (40.6)	168 (65.1)	<0.001
Ischemic cardiovascular disease	18 (17.0)	69 (26.7)	0.047
Chronic kidney disease	25 (23.6)	93 (36.0)	0.021
Hyperthyroidism	6 (5.7)	11 (4.3)	0.566
Lifestyle-related factors†	79 (74.5)	137 (53.1)	<0.001

A comparison of the thromboembolic risk profile between the two groups was performed using Welch's t-test for continuous variables and the Pearson chi-square test for categorical variables. The analysis demonstrated a significantly higher mean CHA₂DS₂-VASc score among non-tribal patients than tribal patients (2.38 ± 1.66 vs. 1.86 ± 1.45; p = 0.004). Consequently, a significantly greater proportion of non-tribal patients met guideline-based criteria for long-term oral anticoagulation (192/258, 74.4% vs. 67/106, 63.2%; p = 0.032). Although the prevalence of prior stroke/TIA and left atrial thrombus was numerically more frequent among tribal patients, these differences did not reach statistical significance. Among patients receiving oral anticoagulation, the choice of oral anticoagulant therapy (VKAs vs. DOACs) was comparable between the two groups (p = 0.090). The comparison of thromboembolic risk profile and anticoagulation practices between tribal and non-tribal patients is presented in Table 5.

**Table 5:** Comparison of thromboembolic risk profile and anticoagulation practices among tribal and non-tribal patients with AF Values are presented as mean ± standard deviation (SD) or n (%). P-values for continuous variables were calculated using Welch's independent-samples t-test, whereas categorical variables were compared using the Pearson chi-square test. *Patients requiring anticoagulation were classified according to CHA₂DS₂-VASc score [11] and valvular disease status based on the 2020 ESC Guidelines [1]. †Percentages for VKAs and DOACs use were calculated among patients receiving oral anticoagulation. Abbreviations: VKAs, vitamin K antagonists; DOACs, directly acting oral anticoagulants; AF, atrial fibrillation; TIA, transient ischemic attack; SD, standard deviation

Characteristic	Tribal (n=106)	Non-tribal (n=258)	p-value
CHA₂DS₂-VASc score (mean ± SD)	1.86 ± 1.45	2.38 ± 1.66	0.004
Prior stroke/TIA, n (%)	23 (21.7)	37 (14.3)	0.086
Left atrial thrombus, n (%)	17 (16.0)	26 (10.1)	0.109
Patients requiring anticoagulation* n (%)	67 (63.2)	192 (74.4)	0.032
VKAs, n (%) †	37 (55.2)	83 (43.2)	0.090
DOACs, n (%) †	30 (44.8)	109 (56.8)	0.090

## Discussion

To our knowledge, this is among the first hospital-based studies from Eastern India to directly compare the clinical characteristics of AF between tribal and non-tribal populations. Although the two groups exhibited a broadly similar demographic profile, important differences were observed in disease presentation and associated risk factors. Tribal patients more frequently presented with first-diagnosed AF, higher mean ventricular rates, lower LVEF, a higher proportion of patients with heart failure symptoms at presentation, and a greater burden of lifestyle-related risk factors, whereas non-tribal patients exhibited a significantly higher prevalence of palpitations at presentation, cardiometabolic disorders, CKD, higher mean CHA₂DS₂-VASc scores, and a greater proportion eligible for long-term oral anticoagulation therapy. Despite these differences, the prevalence of RHD, left atrial enlargement, heart failure, left atrial thrombus, and patterns of oral anticoagulant use remained largely comparable between the two populations.

Perhaps the most important finding of the present study was the significantly higher proportion of first-diagnosed AF among tribal patients. While previous Indian AF registries have comprehensively described the clinical profile and management of AF, none have specifically compared tribal and non-tribal populations [4,9]. The higher frequency of AF first documented at the index hospitalization may reflect differences in prior detection and healthcare contact. Delayed recognition is one possible explanation; however, incident AF and incomplete prior documentation cannot be excluded [5,6]. This interpretation is further supported by the higher ventricular rates at presentation, lower LVEF, and the significantly higher prevalence of heart failure symptoms at presentation observed among tribal patients. These findings highlight the potential value of targeted awareness initiatives and opportunistic AF screening in underserved tribal communities, consistent with contemporary guideline recommendations [1].

Another finding of the present study worth noting was the distinct clinical profile associated with AF in the two populations. Non-tribal patients had a significantly higher burden of cardiometabolic disorders, ischemic cardiovascular disease, and CKD, representing the predominant clinical substrate for AF, consistent with the established risk factor profile reported in large observational studies [1,9,12]. In contrast, tribal patients demonstrated a significantly greater burden of lifestyle-related risk factors, particularly chronic alcohol consumption, smoking, and tobacco use. These findings suggest that, despite a similar demographic profile, the factors predisposing to AF may differ between the two populations, likely reflecting differences in lifestyle exposures and the ongoing epidemiological transition.

Echocardiographic assessment further highlighted differences in the structural phenotype of AF between the two groups. Tribal patients had significantly lower LVEF than non-tribal patients, despite similar left atrial dimensions and a comparable prevalence of heart failure. The lower LVEF may, in part, reflect the greater burden of lifestyle-related risk factors, particularly chronic alcohol consumption, a well-recognized contributor to myocardial dysfunction and AF [8]. Conversely, the comparable prevalence of heart failure despite higher LVEF among non-tribal patients may reflect differences in the underlying heart failure phenotype, potentially including a greater contribution from preserved left ventricular systolic function in the context of their higher cardiometabolic burden. This hypothesis merits further evaluation. The absence of significant differences in left atrial size further suggests that atrial structural remodeling was broadly comparable between the two populations. LA/LAA thrombus was assessed on TTE in all patients, while TEE was additionally performed in patients with persistent clinical suspicion, suspected thrombus, or inadequate visualization on TTE. Therefore, although all documented thrombi were confirmed by TEE, the comparison of thrombus prevalence should be interpreted in the context of this selective use of confirmatory TEE. These findings indicate that ventricular dysfunction, rather than differences in atrial remodeling, may have contributed to the more symptomatic presentation observed among tribal patients. This observation is consistent with previous Indian studies identifying left ventricular systolic dysfunction as an important clinical substrate for AF, particularly among younger patients [10].

Despite differences in cardiometabolic and lifestyle-related risk profiles, the prevalence of RHD remained comparable between tribal and non-tribal patients. This finding challenges the traditional perception that AF among tribal populations is predominantly rheumatic in origin. The comparable prevalence of RHD in the two populations may reflect the persistently high burden of RHD in Eastern India, particularly among socioeconomically disadvantaged populations, where limited access to primary healthcare and delayed diagnosis remain areas of concern [4,5]. Similar observations have been reported in the Indian Heart Rhythm Society-Atrial Fibrillation (IHRS-AF) registry, which demonstrated that although RHD remains an important cause of AF in India, non-rheumatic etiologies are increasingly prevalent [4]. Similar coexistence of traditional cardiovascular risk factors with the persistent burden of RHD has also been observed in contemporary hospitalized AF cohorts from other Asian populations [12]. Comparable trends have also been described in sub-Saharan Africa, where the persistent burden of RHD coexists with a rising prevalence of cardiometabolic risk factors [13,14]. Collectively, these findings highlight the dual epidemiology of AF in this region and emphasize the need for prevention strategies that address the persistent RHD burden along with the growing burden of non-communicable cardiometabolic diseases.

Non-tribal patients demonstrated a significantly higher thromboembolic risk, reflected by higher mean CHA₂DS₂-VASc scores and consequently a greater proportion of patients eligible for long-term oral anticoagulation therapy. This finding is consistent with their greater burden of cardiometabolic comorbidities, which constitute the principal determinants of thromboembolic risk in AF [1,9]. Despite these differences, the prevalence of previous stroke/TIA and left atrial thrombus remained comparable between the two groups, suggesting that conventional clinical risk scores may not fully account for thromboembolic burden in this setting. Importantly, patterns of oral anticoagulant use, including the proportions receiving VKAs and DOACs, were also comparable. The similar prevalence of RHD in both groups likely contributed to this finding, as patients with rheumatic mitral stenosis require long-term VKA therapy irrespective of their CHA₂DS₂-VASc score [1]. These findings indicate that anticoagulant prescribing practices in our cohort were largely driven by the underlying AF substrate and contemporary guideline recommendations rather than tribal status or ethnicity [1].

Our findings from the present study have practical implications for AF care in Eastern India. Although tribal and non-tribal patients shared several clinical characteristics, the differences observed in disease presentation and associated risk factors suggest that preventive strategies should be adapted to the needs of each population. Improving awareness, access to primary healthcare, and opportunistic screening may help identify AF earlier in tribal communities, where patients frequently present with newly diagnosed disease and more advanced symptomatic manifestations. In non-tribal patients, greater emphasis on controlling cardiometabolic risk factors is likely to have more impact on AF prevention and long-term outcomes. Overall, our findings highlight the importance of developing locally relevant, population-specific strategies for AF detection and management instead of relying on a uniform approach.

Limitations

This study has certain limitations. First, its retrospective, single-center design may limit the generalizability of the findings to other regions and healthcare settings. Second, because the analysis was based on hospital medical records, incomplete documentation and residual information bias cannot be completely excluded. Third, the cross-sectional nature of the study precludes inference regarding causal relationships between the observed clinical factors and AF. Fourth, as this was a hospital-based study of admitted patients, selection and referral bias cannot be excluded, and the observed differences may partly reflect differences in healthcare-seeking behavior, referral patterns, or hospitalization thresholds between the groups. Finally, as the analyses were unadjusted, residual confounding due to differences in comorbidities and other clinical characteristics between the groups cannot be excluded; therefore, the observed associations should be considered exploratory. Despite the inclusion of patients over a 36-month period and a relatively large tribal cohort, the single-center design limits the generalizability of the findings. Large-scale, multicenter studies involving diverse tribal and non-tribal populations are needed to validate these findings and establish their broader applicability.

## Conclusions

This study underscores important differences in the clinical phenotype of AF between tribal and non-tribal patients in this hospital-based cohort from Eastern India. Tribal patients exhibited a greater burden of lifestyle-related risk factors, lower LVEF, a higher proportion of first-diagnosed AF, and more frequent heart failure symptoms at presentation. In contrast, non-tribal patients demonstrated a greater burden of cardiometabolic comorbidities and thromboembolic risk, with more patients meeting guideline-based criteria for long-term oral anticoagulation therapy, indicating differences in the clinical and risk profiles of AF between the two groups. The significantly higher proportion of first-diagnosed AF at the index hospitalization among tribal patients may reflect differences in prior healthcare contact or detection; delayed recognition is a possible explanation that requires confirmation in prospective studies. Despite these differences, RHD remained an important contributor to AF in both populations, highlighting the dual burden of rheumatic and non-rheumatic cardiovascular disease in this study population. These findings highlight the distinct clinical and risk profiles observed in tribal and non-tribal patients with AF and support the need for context-specific approaches focusing on earlier detection of AF and comprehensive risk factor modification to improve AF care and reduce its long-term complications.
